# GPDRP: a multimodal framework for drug response prediction with graph transformer

**DOI:** 10.1186/s12859-023-05618-0

**Published:** 2023-12-17

**Authors:** Yingke Yang, Peiluan Li

**Affiliations:** 1 School of Mathematics and Statistics, Henan University of Science and Technology, Luoyang, 471000 China; 2 Longmen Laboratory, Luoyang, 471003 China

**Keywords:** Drug response prediction (DRP), Multimodal deep learning model, Graph transformer, Drug molecular graphs, Pathway activity scores

## Abstract

**Background:**

In the field of computational personalized medicine, drug response prediction (DRP) is a critical issue. However, existing studies often characterize drugs as strings, a representation that does not align with the natural description of molecules. Additionally, they ignore gene pathway-specific combinatorial implication.

**Results:**

In this study, we propose drug Graph and gene Pathway based Drug response prediction method (GPDRP), a new multimodal deep learning model for predicting drug responses based on drug molecular graphs and gene pathway activity. In GPDRP, drugs are represented by molecular graphs, while cell lines are described by gene pathway activity scores. The model separately learns these two types of data using Graph Neural Networks (GNN) with Graph Transformers and deep neural networks. Predictions are subsequently made through fully connected layers.

**Conclusions:**

Our results indicate that Graph Transformer-based model delivers superior performance. We apply GPDRP on hundreds of cancer cell lines’ bulk RNA-sequencing data, and it outperforms some recently published models. Furthermore, the generalizability and applicability of GPDRP are demonstrated through its predictions on unknown drug-cell line pairs and xenografts. This underscores the interpretability achieved by incorporating gene pathways.

**Supplementary Information:**

The online version contains supplementary material available at 10.1186/s12859-023-05618-0.

## Background

Cancer, a highly complex disease, is caused by the interaction of various carcinogenic factors. It significantly impacts global human health and poses a threat to human life. Individuals with the disease exhibit heterogeneity in both genetic and phenotypic aspects, primarily due to the tumor microenvironment's clonal diversity of cancer cells and non-malignant cells with changed phenotypes. This heterogeneity leads to partial or non-responsiveness of certain patients to therapeutic strategies such as chemotherapy, targeted therapy, and immunotherapy during the cancer treatment process [1]. In other words, even when implementing the same therapeutic strategies for patients of the same cancer type, there are still variations in treatment responses, making responses to cancer treatment generally unpredictable. Additionally, it is important to note that not all cancers and anticancer drugs are strongly associated with targetable genetic biomarkers. Therefore, relying solely on the relationship between drug targets or mutation status may be insufficient to predict the efficacy of specific targeted therapies [2, 3]. And implementing targeted therapies without taking drug resistance into account may reduce patient survival rates. Drug resistance may show up as the activation of alternative signaling pathways promoting tumor growth or clonal expansion under the selective pressure induced by treatment [4]. Therefore, drug response prediction (DRP) is critically important in cancer therapy and has become a significant topic in personalized medicine research. Accurate prediction of treatment response assists in designing more effective treatment plans for patients and provides valuable insights for the development of novel disease-inhibiting drugs.

With the rapid development of high-throughput genomics technologies, large-scale pharmacogenomics databases have gradually accumulated. The Cancer Cell Line Encyclopedia (CCLE) [5] provides a platform for systematic study of cell lines. The Genomics of Drug Sensitivity in Cancer (GDSC) [6] is one of the largest public databases, covering information regarding the sensitivity of cancer cells to drugs and related molecular markers. The Cancer Therapeutics Response Portal (CTRPv2) [7] provides extensive data on drug sensitivity. These high-throughput screening research resources collectively form a vast knowledge base [1]. Based on these abundant data resources, numerous researchers have established various DRP models to predict the response of anticancer drugs.

Menden et al. compared anticancer drug sensitivity prediction models constructed using different methods by utilizing two large-scale drug genomics datasets, and demonstrated that genomics can validate the response of specific drugs as an explanatory variable [8]. Ammad-Ud-Din et al. employed a novel nuclear norm-based Bayesian matrix factorization approach that combined drug chemical structure features and genomic characteristics for DRP [9]. Zhang et al. introduced an integrated model to predict drug response in a specified cell line and demonstrated its superiority over the elastic net model [10]. Wang et al. predicted drug response by utilizing the chemical structure of drugs and gene expression profiles, employing a similarity regularized matrix factorization method [11]. Chang et al. proposed the CDRscan, which utilizes cell lines' genomic mutations and molecular fingerprints of drugs for predicting drug efficacy [12]; Sakellaropoulos et al. constructed a model named Precily based on gene expression data for DRP and demonstrated its superior performance over Elastic Net and Random Forest models [13]; Choi et al. introduced an innovative deep neural network model named RefDNN for better drug resistance prediction and biomarker identification related to drug response [14].

Despite significant progress in DRP research, there are some issues worth considering. For instance, most studies represent drugs as strings, which is an unnatural way of representing molecules and may result in the loss of structural information [15]. Additionally, the pathway-specific combinatorial implication (or gene sets) of genes are disregarded, and gene expression levels are treated as independent variables, which may overly emphasize machine learning techniques [16, 17].

To address these issues, we propose GPDRP (Graph and Pathway based Drug response prediction), a novel multimodal deep learning architecture, that can predict drug responses on cell lines by modeling drugs as molecular graphs. In addition, Graph Transformer was combined with Graph Isomorphism Network (GIN) to improve the capacity for more precise DRP. We compared GPDRP with two recently published works: Precily [18], which represents drug moleculars using simplified molecular-input line-entry system (SMILES) strings, and GraTransDRP [19], encoding cell lines’ genomic and epigenomic characteristics through one-hot encoding. Our approach performs better considering root mean square error (RMSE) and Pearson correlation coefficient (PCCs), according to experimental results. Also, by applying GPDRP to 15,094 drug-cell line pairs lacking response values and xenograft datasets, we demonstrated the potential of the model to predict unknown drug-cell line pairs, as well as the applicability of the model and interpretability using gene pathway scores. The primary contributions of GPDRP include:

1. We integrate the drug molecular graph with gene pathway activity score, leveraging the strengths of both types of data to enhance the predictive power of our model.
2. We introduce GPDRP, a novel multimodal framework for DRP, which leverages Graph Convolutional Networks in conjunction with Graph Transformer and deep neural networks. The performance of GPDRP is demonstrated using the CCLE/GDSC dataset, and it outperforms two recently published models, Precily and GraTransDRP.
3. GPDRP demonstrates the potential to predict unknown drug-cell line pairs. It was utilized to predict the pairs that were missing from the GDSC, and some published works were located and discussed that supported our predictions.
4. GPDRP exhibits excellent applicability. We applied it to predict the LNCaP xenograft dataset and provided explanations based on gene activity pathway scores.

## Results

### Performance comparison on the CCLE/GDSC dataset

**Fig. 1:**
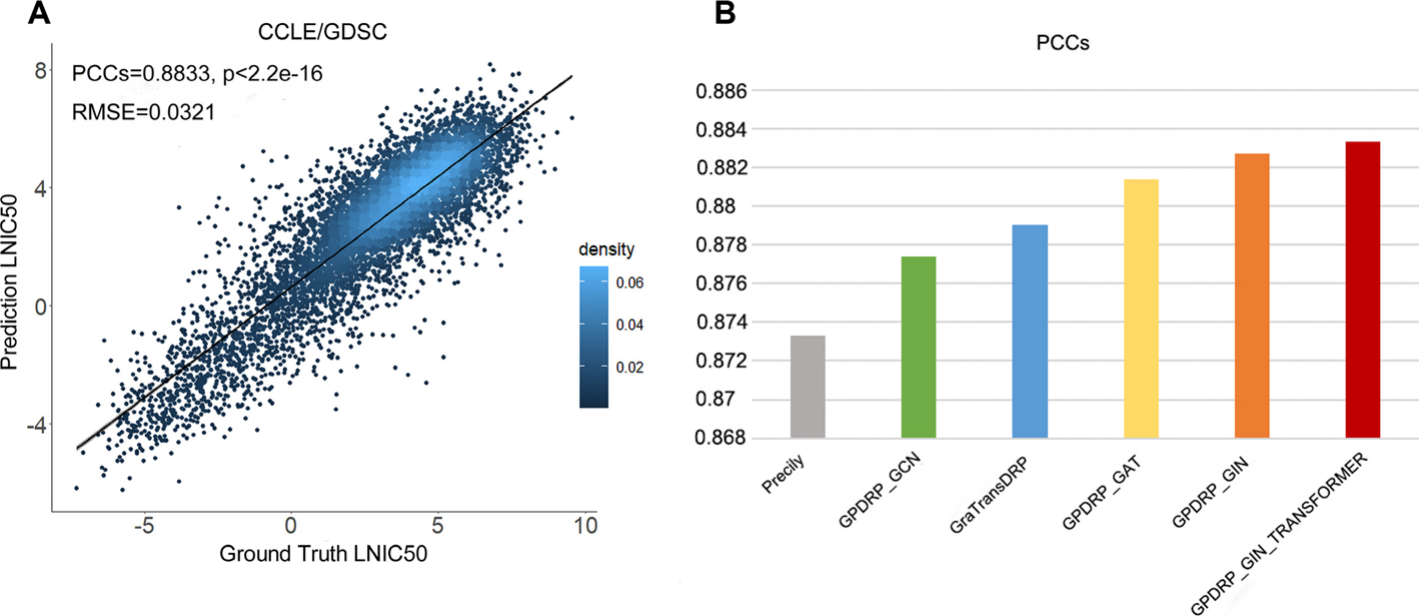
Performance comparison. **A** Scatter plot demonstrating the performance of GPDRP across all drug-cell line pairs in the CCLE/GDSC test data. P-value was calculated using a two-sided t-test. **B** Barplot shows the Pearson’s correlation coefficients (PCCs) for different models

To assess GPDRP's prediction accuracy, we trained the model using the CCLE/GDSC dataset and employed the same data splitting strategy as in Precily [18]. We separated the dataset according to the cell lines, making sure that the test, validation, and training sets did not share any cell lines. Of the total drug-cell line pairs (80,056), we randomly selected 90% (72,156) for the dataset, with 80% of cell lines allocated to the training set and 10% to the validation set for hyperparameter tuning. The remaining 10% (7900) of the pairs were designated for the testing set. The test results revealed a PCCs value of 0.8833 and a RMSE value of 0.0321 in the best model as shown in Fig. 1.

We then compared GPDRP with some recently published models. For methods relying on the identical dataset, PCCs and RMSE were computed. The performance is shown in Fig. 1B and Table 1. Evidently, our model GPDRP outperforms Precily and GraTransDRP for almost all graph convolutional networks. Among three GNN models: Graph Convolutional Networks (GCN), Graph Attention Networks (GAT), and GIN, the GIN model performed the best, achieving a PCCs of 0.8827. This illustrates GIN's potential for graph representation and lends credence to the idea that GIN is one of the most potent GCN models [20]. Therefore, we considered combining GIN with the Graph Transformer, resulting in the best PCCs of 0.8833 and the best RMSE of 0.0321.

**Table 1:** The performance comparison of PCCs and RMSE on the GDSC/CCLE dataset (the best performance is in bold)

Model	PCCs	RMSE
Precily [18]	0.8733	1.3773
GraTransDRP [19]	0.8790	0.0333
GPDRP_GCN	0.8774	0.0325
GPDRP_GAT	0.8814	0.0322
GPDRP_GIN	0.8827	0.0323
GPDRP_GIN_TRANSFORMER	**0.8833**	**0.0321**

### Prediction of responses for unknown drug-cell line pairs

**Fig. 2:**
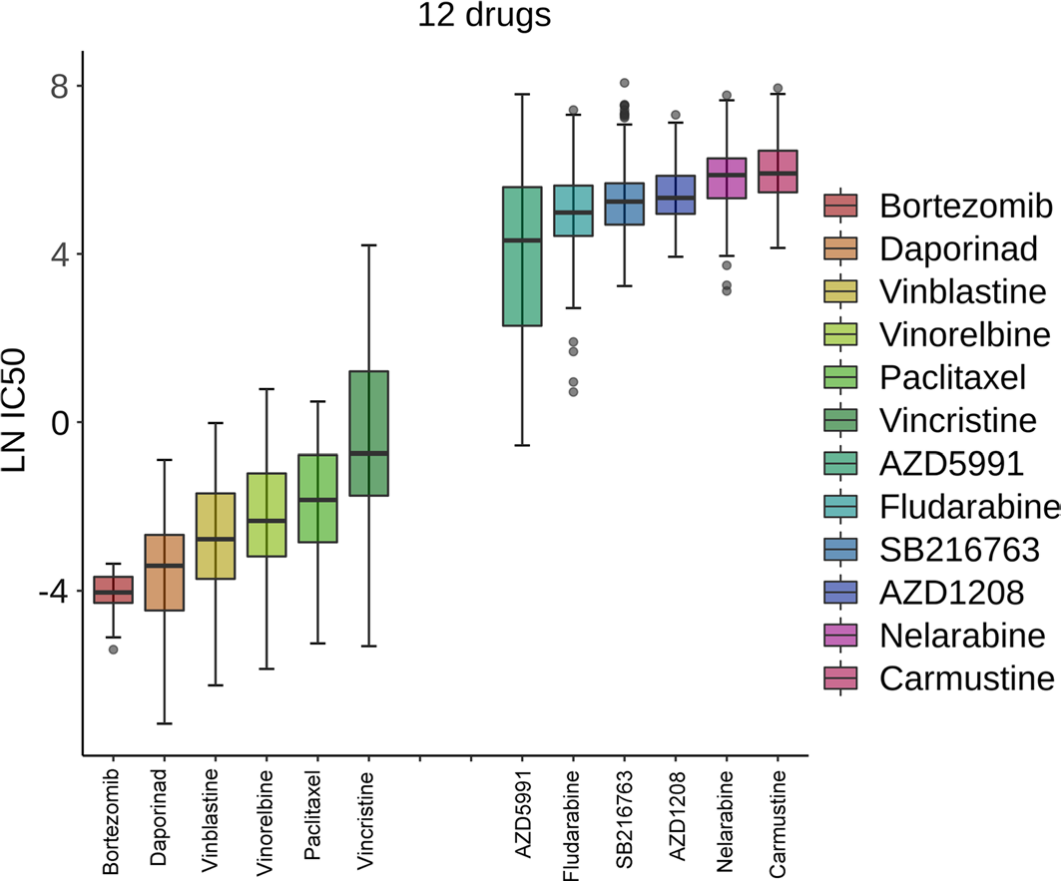
Box plot of predicted LN IC50 values for unknown response pairs. The drugs are arranged based on the median of their predicted LN IC50 values for cell lines. The horizontal axis denotes the drug names, and the vertical axis denotes their LN IC50 values with cell lines. The top 6 drugs with the lowest median LN IC50 values indicate that they may be the most effective drugs, while 6 drugs with the highest median LN IC50 values suggest that they may be the most ineffective drugs

In this part, we used the optimal model, GPDRP_GIN_TRANSFORMER, to predict the response for the processed 15,094 drug-cell line pairs lacking response values (see Additional file 1: Table S3). All the prediction results are provided in Additional file 1: Table S4. The predicted LN IC50 values for the unknown response pairs grouped by drug are displayed in Fig. 2 using a box plot. Drugs are sorted by the median of their distributions, with each drug's box representing the numerical distribution of values associated with its corresponding cell lines. The figure displays six drugs with the highest values and six drugs with the lowest medians. As the true values for these unknown response pairs are unavailable, the accuracy of our prediction is determined by works as follows.

The LN IC50 is the logarithm of the concentration IC50 at which a drug inhibits biological activity. A smaller value indicates greater sensitivity of the cell lines to the drug, indicating its effectiveness. Our predictions identified the top six most effective drugs as Bortezomib, Daporinad, Vinblastine, Vinorelbine, Paclitaxel, and Vincristine. It is noteworthy that Bortezomib, Vinblastine, Paclitaxel, and Vincristine were also identified as potentially effective drugs in the pioneering model proposed by Liu et al. [21].

Our analysis identified Bortezomib as the most potent drug. Bortezomib has demonstrated extensive antitumor activity and has been shown to enhance the efficacy of various chemotherapeutic drugs [22]. Its capacity to sensitize cell lines to numerous other drugs was noted in a study by Friedman et al. [23]. Bortezomib, the initial proteasome inhibitor authorized for the treatment of malignant diseases, is approved for addressing multiple myeloma and mantle cell lymphoma. It has shown positive clinical outcomes as a standalone treatment or as part of a combination therapy, enhancing the effects of chemotherapy/radiation or overcoming drug resistance [24]. Notably, in our predictions, the DOHH2 cell line exhibited the highest sensitivity to Bortezomib among all the unknown drug-cell line combinations. DOHH2, a human non-Hodgkin lymphoma cell line, is frequently used in lymphoma research, and there is evidence supporting Bortezomib's potential in treating non-Hodgkin lymphoma [25].

Daporinad, a potential small molecule compound, exhibits anti-tumor and anti-angiogenic properties. It binds to and inhibits nicotinamide phosphoribosyltransferase (NMPRTase), thereby suppressing the biosynthesis of nicotinamide adenine dinucleotide (NAD+) from nicotinamide (vitamin B3). This activity has the potential to exhaust energy reserves in metabolically active tumor cells and trigger apoptosis. Furthermore, Daporinad may hinder the production of vascular endothelial growth factor (VEGF) in tumor cells, thereby inhibiting tumor angiogenesis. Daporinad has been clinically tested for treating melanoma, cutaneous T-cell lymphoma, and B-cell chronic lymphocytic leukemia [26].

Vinblastine is employed in treating various cancers, including breast cancer, testicular cancer, lymphoma, neuroblastoma, Hodgkin's and non-Hodgkin's lymphoma, as well as fungal infections, histiocytosis, and Kaposi sarcoma [27]. Research by Brugie’res et al. suggested that vinblastine might be effective in treating relapsed anaplastic large cell lymphoma, leading to durable remissions [28]. Vinorelbine, another vinca alkaloid drug, is frequently employed in cancer therapy, encompassing non-small cell lung cancer and breast cancer [29]. Vincristine has maintained a steady role in cancer therapy research, being an integral part of anti-cancer treatment [30].

Paclitaxel, often referred to as an 'anti-cancer superstar,' is a naturally occurring secondary metabolite extracted and purified from the bark of the yew tree, Taxus brevifolia. It has been clinically validated to possess superior anti-tumor properties and is widely used in treating malignancies such as breast cancer, ovarian cancer, and gastric cancer. It is one of the most frequently used chemotherapeutic drugs in clinical practice [31].

The six least effective drugs identified in our study are AZD5991, Fludarabine, SB216763, AZD1208, Nelarabine, and Carmustine. A literature review revealed that these drugs are typically used in combination therapies. For example, Nelarabine is used to treat relapsed or refractory T-cell acute lymphoblastic leukemia (T-ALL) and T-cell lymphoblastic lymphoma (T-LBL) following the failure of at least two previous treatment regimens [32]. Fludarabine can have significant side effects, and careful monitoring of hematologic and non-hematologic toxicities is recommended when used as an anti-cancer drug.

In conclusion, our method has shown exceptional performance in predicting drug responses for unknown drug-cell line pairs, thereby confirming the accuracy and practicality of GPDRP. This allows us to better understand the effects of drugs on specific cell lines, offering robust support for drug development and the creation of personalized treatment strategies.

### Predictions in LNCaP xenografts

Patient-Derived Xenografts (PDXs) are widely used in vivo tumor models to investigate therapeutic responses and forecast drug responses in cancer patients sharing analogous traits. In our study, we applied the GPDRP method to analyze the GSE211856 dataset, which was obtained from the NCBI GEO database (www.ncbi.nlm.nih.gov/geo/). This dataset comprises bulk RNA-seq data from an extensively annotated study on the progression of prostate cancer, focusing on the responsiveness and development of resistance to AR-targeted therapies. Androgens are required for the establishment and early growth of LNCaP xenograft tumors in male mice (pre-castration group, PRE-CX). Castration reduces androgen receptor (AR) activity and tumor growth (post-castration group, POST-CX). This initial sensitivity to castration, however, consistently progresses to castration resistance (castration-resistant prostate cancer, CRPC). Further treatment of CRPC with the AR targeting drug enzalutamide (ENZ) produces an initial therapeutic reaction (ENZ Sensitive, ENZS), but resistance develops over time (ENZ Resistant, ENZR). The dataset includes a total of 54 samples, encompassing multiple biological replicates for each condition and treatment group, as summarized in Table 2.

**Table 2:** GSE211856 dataset overview

Sample	Sample Size	Group
PRE-CX	9	Condition
POST-CX	8	Condition
CRPC	10	Condition
ENZS	12	treatment
ENZR	15	treatment

**Fig. 3:**
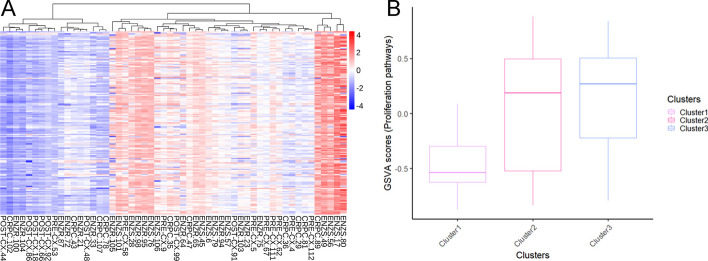
Predictions and analysis in LNCaP xenografts. **A** Heatmap represents the predicted LN IC50 values of 173 drugs across the 54 samples, where lower LN IC50 values are indicated by bluer color bars, indicating greater sensitivity of the predicted samples to the drugs. The samples were grouped based on the Euclidean distance. **B** Boxplots showing the distribution of GSVA scores of proliferation-related pathways (n = 12) across three clusters (n = 12, n = 25 and n = 17 samples from cluster 1, cluster 2 and cluster 3, respectively)

**Fig. 4:**
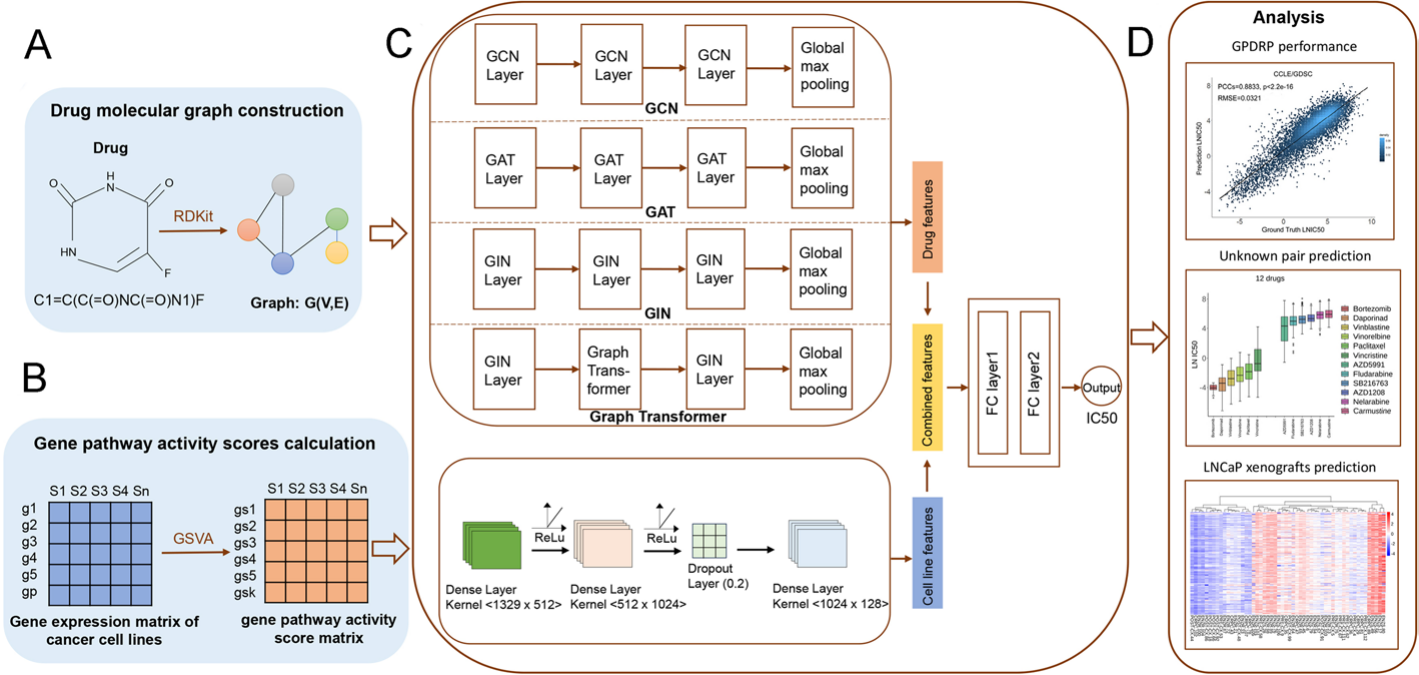
Illustration of the predictive analysis workflow of GPDRP. **A** Drug molecular graph construction. The structure information of drugs was collected from PubChem and we represented drugs as molecular graphs using RDKit. **B** Gene pathway activity scores calculation. For the cancer cell lines obtained from CCLE, we computed pathway activity scores for canonical pathways using GSVA. **C** Two subnetworks for learning drug features and cell line features respectively. GPDRP took molecular graphs of drugs and gene pathway activity scores of cell lines as inputs to the drug subnetworks and cell line subnetworks, respectively. The two representations are then concatenated and put through two FC layers to predict the response. **D** Results and downstream analysis of this work. Including performance comparison, prediction of unknown drug-cell line response and predictions in LNCaP xenografts

To predict drug responses, we used the GPDRP_GIN_TRANSFORMER model trained on the CCLE/GDSC dataset. By applying this model to the 54 samples (see Additional file 1: Table S5), we obtained the predicted sensitivity of 173 drugs on LNCaP xenograft tumor samples, as depicted in Fig. 3. As our response values are continuous and Z-score normalized with a mean of 0 and a standard deviation of 1, we employed Euclidean distance for clustering analysis to enable comparison on a consistent scale. Figure 4 reveals the division of the 54 samples into three main clusters. We summarized the samples with the highest predicted values as Cluster 1, shown as the most red-colored region in Fig. 3A. This cluster exhibits the strongest drug resistance, indicating the lowest drug sensitivity, and is mainly composed of tumor samples treated with ENZ (a total of 12 samples, with 7 ENZS and 3 ENZR). Conversely, we summarized the samples with the lowest predicted values as Cluster 3, shown as the most blue-colored region in the figure, which demonstrates the highest sensitivity to the 173 drugs. Notably, ENZR samples are distributed across all three clusters, suggesting heterogeneity in treatment outcomes and implying that ENZ resistance may involve different underlying mechanisms, potentially involving interactions with stromal components in the tumor microenvironment [18].

To further elucidate the clustering results, we focused on pathway activity scores related to cell proliferation, as shown in Fig. 3B. The pathways related to cell proliferation that we utilized are provided in Additional file 1: Table S6. Box plots were utilized to illustrate the variances in pathway activity scores among the three clusters. Cluster 1 exhibited the lowest pathway activity scores in cell proliferation-related pathways, which may account for the lowest sensitivity to drug responses in this cluster. Conversely, Cluster 3 displayed the highest pathway activity scores, indicating a higher proliferation index, thereby explaining the increased sensitivity to drug responses in this cluster. Therefore, the use of gene activity scores makes the model results more interpretable.

## Discussion

Accurate prediction of drug response in cancer cells is pivotal for personalized oncology. This work introduces GPDRP, a multimodal deep learning framework leveraging the Graph Transformer architecture to forecast the response to cancer treatment, utilizing information from both drug molecular graphs and gene pathway activity. We employed four GNN variants: GCN, GAT, GIN and Graph Transformer with the combination of GIN, used for learning drug features. Subsequently, the drug-cell line pairs were used to predict LN IC50 values. Notably, our model combines drug molecule graphs with gene pathway activity scores, outperforming some recently published methods in terms of performance comparisons based on RMSE and PCCs.

The experimental results indicate that GPDRP outperforms in terms of RMSE and PCCs. Through performance comparison, we believe that representing drugs using graphical structures may preserve the essence of their chemical structures, making it more appropriate than using strings. In this experiment, GPDRP_GIN_TRANSFORMER demonstrated superior performance, possibly due to the addition of a Graph Transformer layer. Firstly, the multi-layer feature extraction capabilities of GIN and Graph Transformer complement each other. GIN excels in capturing local neighborhood features, while the Graph Transformer layer effectively captures long-range dependencies and global relationships among nodes through its self-attention mechanism. The combination of these two layers enables the model to learn more comprehensive and informative graph structure features. Secondly, the integration of local and global information enhances the model's representational power. GIN's neighborhood aggregation process may overlook long-distance relationships, which can be effectively addressed by the Graph Transformer layer's ability to capture global dependencies. By incorporating the Graph Transformer layer after the GIN layers, the model achieves a better fusion of local and global information. This integration leverages the strengths of both models, allowing for comprehensive feature extraction, effective combinations of local and global information, and improved generalization abilities on graph data. Furthermore, when predicting responses for drug-cell line pairs with unknown responses in the CCLE/GDSC dataset, we identified Bortezomib, Daporinad, Paclitaxel, and vinca alkaloids as drugs with the lowest response values, highlighting their anti-tumor properties. Conversely, drugs with the highest response values exhibited lower sensitivity to cancer, illustrating the model's potential to learn from data and predict responses for new drug-cell line pairs. We further demonstrate the applicability of GPDRP in LNCaP xenografts and its interpretability using gene pathway activity scores.

One limitation of GPDRP is the interpretability of the model. We employ GNN to learn the latent features of drug molecular graphs. While Nguyen et al. [15] demonstrated that GNN can assign significance to clearly defined chemical features automatically without prior knowledge, the majority of the learned latent variables still defy explanation using available descriptors (specific details provided in Additional file 1: Supplementary Materials C). Furthermore, our study solely focuses on cell lines, and when it comes to data splitting based on drug compounds, the model falls short of achieving the anticipated outcomes (specific details provided in Additional file 1: Supplementary Materials D). This may be attributed to the vast chemical space of drug compounds. In the future, we will place a particular emphasis on researching model interpretability and give greater attention to drug-based research to enhance the model's interpretability and improve its effectiveness in predicting drug responses. Additionally, RGCN and RGAT may enhance the predictive capabilities of the model, and we will explore their use to achieve better predictive performance.

## Conclusions

In this paper, we propose a multimodal deep learning model, GPDRP, which enables more accurate prediction of drug responses. By employing drug molecular graphs as the representation of drugs and leveraging GNN with Graph Transformer for feature extraction, this approach may better preserve the structural information of drug molecules, enhancing the model's understanding and predictive capability of drug features. Furthermore, through the incorporation of gene pathway activity scores, GPDRP provides valuable interpretability. The introduction of this model holds significant implications, offering a precise tool for personalized medicine and cancer treatment, and driving advancements in cancer research.

## Methods

We propose a multimodal deep learning architecture, called GPDRP for DRP. The DRP problem is formulated as a regression task, wherein a drug-cell line pair serves as the input and a continuous measurement of the response value LN IC50 of that pair serves as the output. Molecular graphs are used to represent drugs, which allows the model to directly capture atom-to-atom bonds. GPDRP is trained using the Pytorch [33]. Figure 4 illustrates the proposed framework.

### Data acquisition

For comparison purposes, we followed the same procedure as in Precily [18], obtaining 550 CCLE cell lines' bulk RNA-seq gene expression profiles that overlap with the GDSC2 dataset of the GDSC database. The relevant response data was extracted from the GDSC2 dataset. We collected information on drug responses for 173 compounds, and their SMILES notations were retrieved using PubChemPy [34]. Specific data processing is provided in Additional file 1: Supplementary Materials A.

### Drug molecular graph construction

For drug features, we perceive drug compounds as graphs depicting the interactions among atoms. Firstly, 173 molecular compounds' chemical structure data was obtained in terms of a Canonical SMILES using PubChemPy (see Additional file 1: Table S1). Then using the open-source cheminformatics program RDKit [35], we translated the Canonical SMILES into the corresponding molecular graphs and extracted atomic features. We employed a collection of atomic attributes adapted from DeepChem [36] to characterize a node in the molecular graph. Each node is represented as a multidimensional binary feature vector conveying five distinct pieces of information: the atomic symbol, the number of neighboring atoms, the number of neighboring hydrogen atoms, the implicit valence of the atom, and whether the atom is part of an aromatic structure. The presence of a bond between a pair of atoms triggers the establishment of an edge. Consequently, an indirect binary graph, comprising nodes endowed with associated attributes, is constructed for each input Canonical SMILES.

### Gene pathway activity scores calculation

For cell lines features, we used pathway activity scores (see Additional file 1: Table S2). Based on the gene expression matrix, we computed Gene Set Variation Analysis (GSVA) scores using the GSVA [37] R software package, utilizing 1329 gene sets from the Molecular Signatures Database (MSigDB) [38] make up the c2 canonical pathway collection (MSigDB.CP.v.6.1, see Additional file 1: Supplementary), with min.sz set to 5. By calculating GSVA scores, we transformed the gene expression matrix into a GSVA score matrix comprising 1329 pathway activity scores and 550 cell lines. The resulting GSVA score matrix served as the cell line feature matrix. To enhance the convergence and stability of the model, each feature is normalized to the [0,1] range using min–max scaling. For the $k$th cell line on the $i$th pathway, the normalization is performed as follows:

$$\hat{x}_{ik} = \frac{{x_{ik} - \min (x_{i} )}}{{\max (x_{i} ) - min(x_{i} )}},$$

where $x_{ik}$ represents the $k{\text{th}}$ cell line’s pathway activity score on the $i{\text{th}}$ pathway, while $min(x_{i} )$ and $\max (x_{i} )$ respectively denote the minimum and maximum values of pathway $i$ across all cell lines.

### Processing of the response variable

After processing the drug and cell line data, we obtained 95,150 drug-cell line pairs. There were 15,094 pairs for which corresponding response values LN IC50 are not available in the GDSC database, and 80,056 pairs have LN IC50. Therefore, we used these 80,056 pairs along with their corresponding response values for model training and testing. Additional file 1: Supplementary Materials B contained the dataset's summary statistics. In addition, we scaled the drug response values LN IC50 values within the range of (0,1) to facilitate the training. For a given LN IC50 value $x$, the actual value is $y = e^{x}$, and the subsequent function is employed to normalize $y$: the actual

$$\hat{y} = \frac{1}{{1 + y^{ - 0.1} }},$$

in order to distribute the result more evenly on (0, 1),the parameter value of $- 0.1$ is typically selected when y is very small ($< 10^{ - 3}$) [8].

### Two subnetworks for drugs and cell lines

Conceptually, GPDRP can be viewed as a multimodal deep learning model comprising two subnetworks designed for processing drug and cell line features.

For drug features, graph convolutional networks may be well-fitting for DRP because a graph is used to represent the drug's molecular structure. In light of the widespread utilization of Graph Convolutional Networks (GCN) in the context of drug response prediction [19, 39, 40], we investigated four graph convolutional models, including Graph Convolutional Networks (GCN) [41], Graph Attention Networks (GAT) [42], Graph Isomorphism Network (GIN) [43] and Graph Transformer with the combination of GIN, all of which we described as follows. Following the GNN, a fully connected layer (FC layer) was additionally utilized to transform the outcome into 128 dimensions.

For cell line features, we used pathway activity scores and employed deep neural networks (DNN) with three hidden layers to learn features. The DNN architecture consisted of an input layer succeeded by three dense layers with sizes of 512, 1024, and 128, respectively, using Rectified Linear Unit (ReLU) as the activation function. The architecture incorporated a dropout layer with a rate set to 0.2 after the second dense layer to prevent overfitting. Then the output was flattened to a 128-dimensional vector. Subsequently, the 256-dimensional vector, encompassing both drug and cell line features, traversed two FC layers to predict drug response, with 1024 and 128 nodes respectively. The LN IC50 was used to quantify GPDRP output and indicated how well a medication inhibited the growth of a particular cancer cell line. A high level of drug efficacy was indicated by small IC50 values, which suggested that the drug was sensitive to the corresponding cancer cell line [28]. The hyper-parameters utilized in our experiments are listed in Table 3. They were chosen on the basis of prior research experience rather than tuned.

**Table 3:** Hyper-parameters for different graph neural network variants used in our experiments

Hyper-parameters	Setting
Activation	ReLu
Optimizer	Adam
Learning rate	0.0001
Dropout	0.2
GCN Layers	3
GAT Layers	3
GIN Layers	3
GIN_TRANSFORMER Layers	3
DNN Layers	3

### Graph convolutional networks (GCN)

Predicting a continuous value that represents the LN IC50 of drug sensitivity in cell lines is our main goal in this work. We employ GCN to learn about each drug graph representation. Formally, $G = (V,E)$ denotes the graph of a given drug, where $V$ is the set of $N \in R$ nodes, each characterized as a C-dimensional vector, and $E$ represents the set of edges, which is denoted by an adjacency matrix $A \in R^{N \times N}$. A node feature matrix $X \in R^{N \times C}$ and an adjacency matrix $A$ are inputs to the multi-layer GCN. Then it generates a node-level output $Z \in R^{N \times F}$ with $F$ denoting the quantity of features each node output. A normalized form is employed to express the propagation rule as follows:

$$H^{(l + 1)} = \sigma (\tilde{D}^{{ - \frac{1}{2}}} \tilde{A}\tilde{D}^{{ - \frac{1}{2}}} H^{(l)} W^{(l)} ),$$

where $\tilde{A} = A + I_{N}$, $\tilde{D}$ is the graph diagonal degree matrix. And $\sigma$ is an activation function, $H^{(l)} \in R^{N \times C}$ is the $l{\text{ - th}}$ layer’s activation matrix, $H^{(0)} = X$, $W$ is learnable parameters.

Three consecutive GCN layers are used in our GCN-based model, and the ReLU function is applied after each layer. After the last GCN layer, a global max pooling layer is incorporated to capture the representation vector of the entire graph, which is then combined with the representation of the cell line to predict the response value.

### Graph attention networks (GAT)

The GAT is constructed through the layering of a graph attention layer. It introduces an attention-based structure to acquire latent node representations within a graph, employing a self-attention mechanism. The GAT layer uses a weight matrix $W$ to apply a linear transformation to each node in a set of graph nodes that it receives as input. And the attention coefficients between node $i$ and its first-order neighbors $j$ are computed in the graph as

$$\alpha (W_{{x_{i} }} ,W_{{x_{j} }} ).$$

Subsequently, these attention coefficients undergo normalization through a softmax function and are employed to calculate the output features for the nodes as

$$\sigma \left( {\sum\limits_{{j \in {\rm N}(i)}} {\alpha_{ij} W_{{x_{j} }} } } \right),$$

where $\sigma ( \cdot )$ is a non-linear activation function and $\alpha_{ij}$ are the normalized attention coefficients.

Our GAT-based model consists of three GAT layers activated by a ReLU function, followed by a global max pooling layer to obtain the graph representation vector. Multi-head-attentions are used for the first GAT layer, with the number of heads set to ten. The second and third GAT's output features are limited to 128.

### Graph Isomorphism network (GIN)

The GIN is a recent approach believed to attain optimal discriminative capability within graph neural networks. It employs a multi-layer perceptron (MLP) model for updating the node features as

$$MLP\left( {(1 + \mu )x_{i} + \sum\limits_{{j \in {\rm N}(i)}} {x_{i} } } \right),$$

where $\mu$ is either a learnable parameter or a fixed scalar, $x$ is the node feature vector, and ${\rm N}(i)$ is the set of nodes neighbor to $i$.

Three GIN layers are stacked in our GIN-based model to build architecture, with a batch normalization layer added after each layer. A global max pooling layer is added for aggregating a graph representation vector, similar to previous architectures.

### Graph transformer

GCN and GAT are designed to learn on homogeneous graphs. GIN updates node representations by utilizing only the features of local neighboring nodes, which may result in insufficient capture of global information. In contrast, Transformer can facilitate better feature learning for more generalized drug graphs. With its self-attention mechanism, Transformer can simultaneously consider the information from all nodes in the graph, enabling a more effective integration of global information.

Drug graph $G = (V,E)$ has a set of node type $T^{v}$, and a set of edge type $T^{e}$. There is an adjacency tensor $A \in R^{N \times N \times K}$, where $K = \left| {T^{e} } \right|$ and feature matrix $X \in R^{N \times F}$. A meta-path is defined to predict new connections among nodes as

$$A_{P} = A_{t1} \cdots A_{tp} ,$$

where $A_{ti}$ is an adjacency matrix for the $i$th edge type of meta-path. For $A_{ti}$, a soft adjacency matrix $Q$ using $1 \times 1$ convolution is

$$Q = F(A,W_{\phi } ) = \phi (A,soft\max (W_{\phi } )),$$

where $\phi$ is a convolution layer and $W_{\phi } \in R^{1 \times 1 \times K}$. Combining with GCN, node representations are constructed as

$$Z = \left\| {_{i = 1}^{C} \sigma (\tilde{D}_{i}^{ - 1} \tilde{A}_{i}^{(l)} XW)} \right..$$

$Z$ is a function of neighborhood connectivity. Extracting features from the graph poses challenges in determining the node positions because of the inherent characteristics of the graph, the Graph Transformer utilizes Laplacian eigenvectors to address this concern as.

$$\Delta = I - D^{{ - {1 \mathord{\left/ {\vphantom {1 2}} \right. \kern-0pt} 2}}} AD^{{ - {1 \mathord{\left/ {\vphantom {1 2}} \right. \kern-0pt} 2}}} = U^{T} \Lambda U,$$

where $U$ and $\Lambda$ are eigenvectors and eigenvalues, respectively.

In our model, we combined one Graph Transformer layer with two GIN layers to improve feature extraction and prediction accuracy.

### Performance evaluation

Two metrics were utilized to assess the performance of the models: Root Mean Squared Error (RMSE) and Pearson Correlation Coefficient (PCCs). RMSE is calculated as the square root of the mean squared error, representing the average squared difference between the actual and predicted responses. PCCs endeavors to gauge the presence of a linear correlation between two variables. Given $n$ samples, $O$ is the actual response value, and $Y$ is the predicted response value. The actual response walue of $i{\text{th}}$ sample is $o_{i}$, and $i{\text{th}}$ sample’s predicted response value is $y_{i}$. RMSE is calculated as follows:

$$RMSE = \sqrt {\frac{1}{n}\sum\nolimits_{i}^{n} {(o_{i} - y_{i} )^{2} } } .$$

The PCCs of $o_{i}$ and $y_{i}$ is defined as follows:

$$PCCs = \frac{{\sum\nolimits_{i}^{n} {(o_{i} - y_{i} )^{2} } }}{{\sigma_{O} \sigma_{Y} }}.$$

where $\sigma_{O}$ and $\sigma_{Y}$ are the standard deviations of ground-truth $O$ and predicted value $Y$, respectively.

### Supplementary Information

**Additional file 1**. Supplementary materials and tables.

## Data Availability

Publicly available datasets were analyzed in this study. Data supporting the findings of this study are available in Cancer Cell Line Encyclopedia at https://sites.broadinstitute.org/ccle/, Genomics of Drug Sensitivity in Cancer at https://www.cancerrxgene.org/ and NCBI GEO database (accession number GSE211856) at https://www.ncbi.nlm.nih.gov/geo/. The code used for this paper is available on GitHub (https://github.com/yyk124/GPDRP).

## Abbreviations

GPDRP: Graph and gene pathway based drug response prediction method
GNN: Graph neural networks
DRP: Drug response prediction
CCLE: The Cancer Cell Line Encyclopedia
GDSC: The Genomics of Drug Sensitivity in Cancer
CTRPv2: The cancer therapeutics response portal
GIN: Graph isomorphism network
SMILES: Simplified molecular-input line-entry system
PCCs: Pearson correlation coefficient
RMSE: Root mean square error
GCN: Graph convolutional networks
GAT: Graph attention networks
