# Supplementary material for: GPDRP: a multimodal framework for drug response prediction with graph transformer
Source: BMC Bioinformatics. 2023 Dec 17;24:484. doi: 10.1186/s12859-023-05618-0 (PMC10726525; doi:10.1186/s12859-023-05618-0)
Supplement: Supplementary file 1 — Additional file 1. Supplementary materials and tables. [file 12859_2023_5618_MOESM1_ESM.zip › Supplementary file/Supplementary Materials.docx]

**Supplementary Material:**

**GPDRP: A multimodal framework for drug response prediction with Graph Transformer**

**Contents**

[A． Data processing 1](#_Toc149232497)

[B． Overall summary of the CCLE/GDSC dataset 2](#_Toc149232498)

[C． Graphical models discover known drug properties 3](#_Toc149232499)

[D． Data splitting based on drugs (Blind-drug test) 4](#_Toc149232500)

[References 5](#_Toc149232501)

# Data processing

Our final CCLE/GDSC training dataset constituted 80056 drug-cell line pairs, including 173 drugs and 550 cell lines. Each drug has its own Canonical SMILES, and each cell line has 1329 pathway activity scores.

For cell lines, we used pathway activity scores. Firstly, we utilized publicly available TPM (transcript per million) normalized RNA-seq gene expression profiles of 1019 Cancer Cell Line Encyclopedia (CCLE) [1] cell lines. These profiles were quantified using the RSEM (RNA-Seq by Expectation-Maximization) software. Secondly, the corresponding drug response information for the cell lines was sourced from the GDSC2 dataset within the Genomics of Drug Sensitivity in Cancer (GDSC) database [2]. The drug-cell line pairs with multiple LNIC50 measurements are averaged. Thirdly, the RNA-seq gene expression profiles of 550 CCLE cell lines overlapped with GDSC2 dataset was screened for model training. This matrix consisted of 57820 Ensembl Gene IDs. We converted these IDs into official gene symbols using gencode.v19.genes.v7_model.patched_contigs.gtf annotation file. Some individual gene symbols had multiple corresponding Ensembl gene IDs. In such cases, we considered the average expression. At this stage, our expression matrix comprised 54301 genes and 550 cell lines, and the matrix was subjected to log2 transformation with the addition of a pseudo count of 1. Finally, based on gene expression matrix, we computed Gene Set Variation Analysis (GSVA) scores using the GSVA [3] R software package, utilizing the c2 collection of canonical pathways (MSigDB.CP.v.6.1) consisting of 1329 gene sets from the Molecular Signatures Database (MSigDB) [4] with min.sz set as 5. By calculating GSVA scores, we transformed gene expression matrix into a GSVA score matrix comprising 1329 pathway activity scores and 550 cell lines.

For drugs, we obtained drug response information for 192 compounds from the GDSC2 dataset for the 550 cell lines in the CCLE dataset. The chemical structure information for these molecular compounds was retrieved in terms of a simplified molecular-input line-entry system (SMILES) using PubChemPy [5]. However, SMILES were not available for all the molecular compounds. As a result, we ended up with SMILES of 173 compounds for 550 CCLE cell lines.

# Overall summary of the CCLE/GDSC dataset

**Table 1.** Overall summary of the CCLE/GDSC dataset.

| **Cancer** | **#Cell line** | **#Drug** |
| --- | --- | --- |
| ALL | 13 | 156 |
| BLCA | 16 | 156 |
| BRCA | 44 | 171 |
| CESC | 2 | 155 |
| CLL | 2 | 160 |
| COREAD | 41 | 156 |
| DLBC | 11 | 156 |
| ESCA | 22 | 156 |
| GBM | 18 | 156 |
| HNSC | 11 | 156 |
| KIRC | 12 | 156 |
| LAML | 14 | 155 |
| LCML | 9 | 156 |
| LGG | 6 | 155 |
| LIHC | 14 | 156 |
| LUAD | 48 | 156 |
| LUSC | 14 | 156 |
| MB | 3 | 156 |
| MESO | 6 | 155 |
| MM | 14 | 156 |
| NB | 9 | 156 |
| OV | 18 | 156 |
| PAAD | 26 | 156 |
| PRAD | 5 | 155 |
| SCLC | 30 | 156 |
| SKCM | 22 | 156 |
| STAD | 19 | 156 |
| THCA | 8 | 156 |
| UCEC | 8 | 156 |
| UNCLASSIFIED | 85 | 170 |

The table shows the frequency of cell lines (n=550) from the CCLE database and the frequency of tested drugs (n=173) from the GDSC database spanning 29 classified cancer types used for the training dataset.

# Graphical models discover known drug properties

We proposed GPDRP based on drug-target binding affinity prediction research [6] and utilized graph neural networks to abstract each drug's molecular graph into a new latent variable feature vector, collectively representing the drug's structural characteristics using 128 latent variables, but the model faces challenges in terms of interpretability. Although identifying the specific molecular substructures corresponding to each latent variable is not straightforward, the study of [6] demonstrates that it is possible to learn a latent space with overlapping information from known molecular descriptors using matrix regression. They found that 20.19% of the latent space can be explained by known descriptors, with the 'Number of aliphatic OH groups' contributing most to the explained variance. In fact, two latent variables closely correlate with this descriptor: when the number of aliphatic OH groups is high, hidden nodes V58 and V14 tend to have high activations. Figure 1 displays a redundancy analysis of the 128 latent variables regressed with 38 molecular descriptors [6].


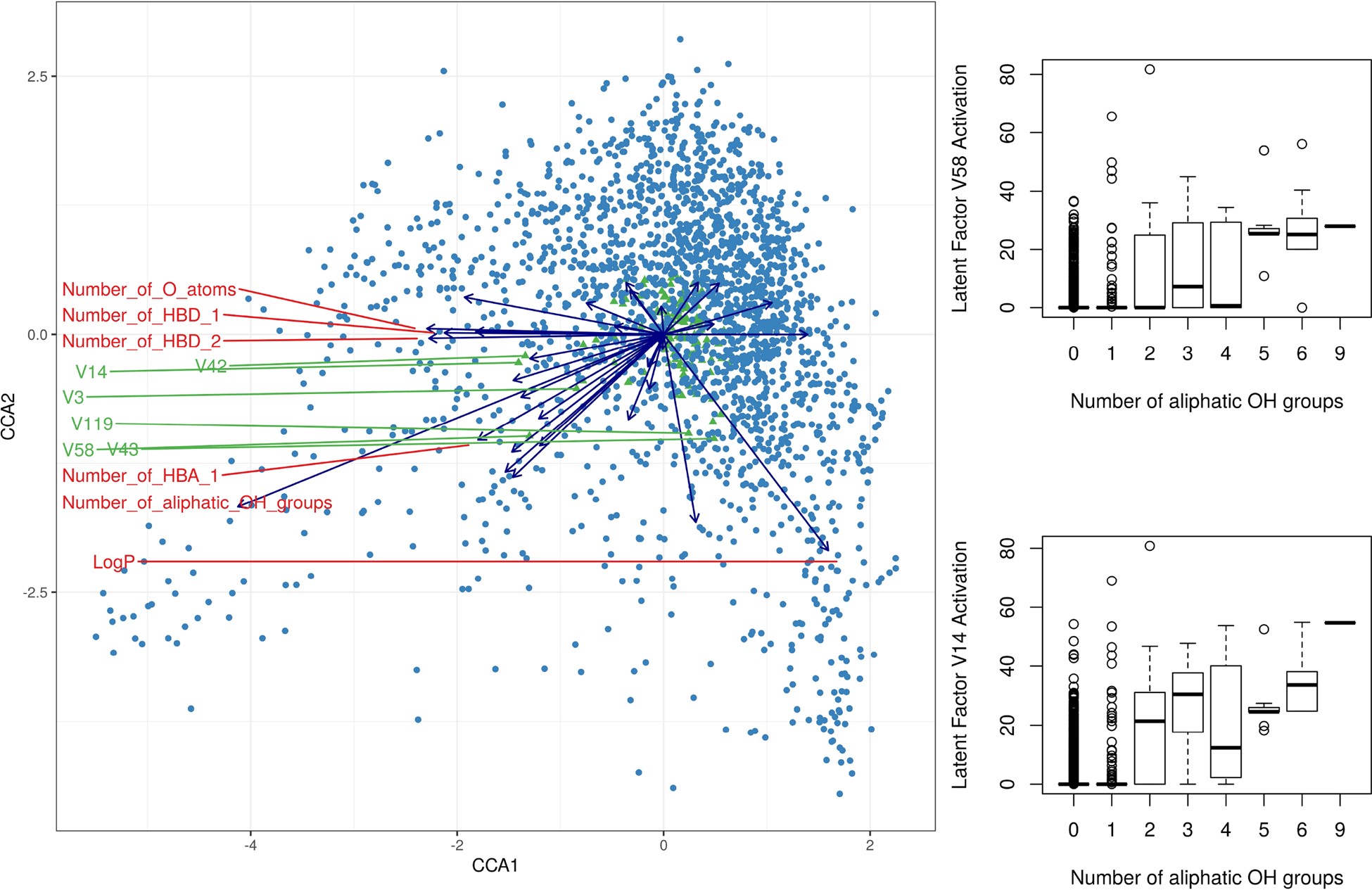


**Figure 1 [6].** The left panel of the figure shows a redundancy analysis triplot for the 128 drug latent variables regressed with 38 JoeLib molecular descriptors. The blue dots represent drugs, the green dots represent latent variables (the 6 furthest from origin are labelled) and the arrows represent molecular descriptors (the 5 longest are labelled). The right panel of the figure shows the activation of two latent variables plotted against the number of aliphatic OH groups in that drug. These results suggest that the graph convolutional network can abstract known molecular descriptors without any prior knowledge.

This discovery provides some insights into how the graph model 'views' drugs in terms of a set of molecular substructures, even though the majority of the latent space is orthogonal to known molecular descriptors.

# Data splitting based on drugs (Blind-drug test)

In previous experiments, it was possible for the drugs present in the test set to also appear in the training phase. However, there are instances where we need to predict the responses of new drugs, such as newly developed pharmaceuticals. Therefore, we conducted a drug-blind test, aiming to assess the predictive performance of previously unseen drugs. The data were splitting based on drugs. In 90% of the drugs (156/173), we randomly selected their response values for training, with 80% of the drugs used in the training set and 10% in the validation set. The remaining 10% (17/173) of drugs were reserved for the test set. Table 2 displays the predictive performance when using previously unseen drugs for blind testing. Notably, GPDRP_GIN_TRANSFORMER achieved the highest Pearson's correlation coefficients (PCCs) and lowest root mean square error (RMSE).

**Table 2.** Performance in terms of PCCs and RMSE on our dataset in blind-drug test

| **Model** | **PCCs** | **RMSE** |
| --- | --- | --- |
| GPDRP_GCN | 0.4774 | 0.0563 |
| GPDRP_GAT | 0.3814 | 0.0573 |
| GPDRP_GIN | 0.0317 | 0.0768 |
| GPDRP_GIN_TRANSFORMER | **0.6978** | **0.0534** |

Interestingly, the model's generalization performance in drug-blind testing, assessed by common performance metrics, is noticeably worse than that of cell-line blind testing, and in some cases, the model fails to make effective predictions altogether. The lower performance can be attributed to the vast chemical space of drug compounds, posing a challenge to predictive models trained on only a few hundred drugs typically used in conventional drug screening studies. Furthermore, empirical evidence suggests that the diversity of drugs is a major contributor to most of the variation in responses [7], which can explain the performance drop observed in drug-blind analysis.

Models proficient in extrapolating to unknown drugs could be valuable for repurposing non-cancer therapies for cancer indications and developing new drugs for cancer treatment. Therefore, in our future work, we will place a strong emphasis on drug-centric research to design more effective models for predicting drug responses.

# References

[1] Barretina J, Caponigro G, Stransky N, Venkatesan K, Margolin AA, Kim S, G, et al. The Cancer Cell Line Encyclopedia enables predictive modelling of anticancer drug sensitivity. Nature 2012; 483(7391): 603-607.

[2] Yang W, Soares J, Greninger P, Edelman EJ, Lightfoot H, Forbes S, et al. Genomics of Drug Sensitivity in Cancer (GDSC): a resource for therapeutic biomarker discovery in cancer cells. Nucleic Acids Res 2013; 41(Database issue): D955-D961.

[3] Hänzelmann S, Castelo R, Guinney J. GSVA: gene set variation analysis for microarray and RNA-seq data. BMC bioinformatics 2013; 14: 1-5.

[4] Subramanian A, Tamayo P, Mootha VK, Mukherjee S, Ebert BL, Gillette MA, Paulovich A, Pomeroy SL, Golub TR, Lander ES, Mesirov JP. Gene set enrichment analysis: a knowledge-based approach for interpreting genome-wide expression profiles. Proceedings of the National Academy of Sciences 2005; 102(43): 15545-15550.

[5] Swain, M. PubChemPy: A way to interact with PubChem in Python. (2014).

[6] Nguyen T, Le H, Quinn TP, Nguyen T, Le TD, Venkatesh S. GraphDTA: predicting drug-target binding affinity with graph neural networks. Bioinformatics 2021; 37(8): 1140-1147.

[7] Partin A, Brettin TS, Zhu Y, Narykov O, Clyde A, Overbeek J, Stevens RL. Deep learning methods for drug response prediction in cancer: Predominant and emerging trends. Front Med (Lausanne) 2023; 10:1086097.
